# Surface free energy of polyurethane coatings with improved hydrophobicity

**DOI:** 10.1007/s00396-012-2598-x

**Published:** 2012-02-14

**Authors:** Piotr Król, Bożena Król

**Affiliations:** Department of Polymer Science, Faculty of Chemistry, Rzeszów University of Technology, Al. Powstańców Warszawy 6, 35-959 Rzeszów, Poland

**Keywords:** Polyurethane coatings, Phase structure, Physico-chemical interactions, WAXS, DSC, Surface free energy parameters by van Oss–Good and Owens–Wendt methods

## Abstract

The polarity of polyurethane coats was studied on the basis of the goniometric method for determination of wetting angle values, on the basis of calculated surface free energy (SFE) values by the van Oss–Good and Owens–Wendt methods, and on the basis of polarity measurements with the use of the ^1^H NMR spectra. Test polyurethanes were synthesised in the reaction of methylene diphenyl 4,4′-diisocyanate (MDI) or 3-izocyanatomethyl –3,5,5- trimethylcyclohexyl isocyanate (IPDI) and polyoxyethylene glycols or polyesters poly(ε-caprolactone) diols and poly(ethyleneadipate) diol with different molecular weights, and some diols as chain extenders, in dioxane. The type of raw material was found to significantly affect the phase structure of the obtained polyurethane elastomers and to control physical interactions within those structures, thus influencing the SFE values. Fundamental reduction in the SFE value of a coating below 28 mJ/m^2^ was achieved by the use of 2,2,3,3-tetrafluoro-1,4-butanediol as the urethane prepolymer chain extender.

Polyurethanes, i.e. polymers which are produced in the polyaddition process of diisocyanates and polyols, make a group of generally polar polymers for which the surface free energy (SFE) values do not exceed the level of 40 mJ/m^2^. Improved polarity is required for modern applications of those plastics in materials engineering, predominantly as protective coatings and structural elastomers which mate with metallic elements, and—in case of biomaterials—their surfaces should be much more hydrophobic. The chemical performance of polyurethanes was already demonstrated in earlier reports to be controlled by chain structures of polyurethanes, and in particular, by polar interactions and hydrogen bonds between their soft and hard segments [1, 2]. Which is decisive, however, for the improved hydrophobicity is the chemical constitution and physical nature of the polymer surface [3]. Higher polarity of polyurethane is advantageous, e.g. for its enhanced adhesion when thermoplastic polyurethane elastomer is co-moulded with galvanised steel [4]. The polarity of polyurethanes may be increased within some limits by structural changes which control polar and dispersion interactions between chain structural segments and additional fillers, like activated carbon [5]. Non-metallic mineral particles (CaCO_3_, TiO_2_ and loess) can be incorporated into waterborne polyurethane acrylate to improve surface properties of eco-friendly floor tiles [6]. And on the contrary, the addition of hydroxy-terminated polydimethylsiloxane into soft segments of UV-curable polycarbonate-based polyurethane acrylate dispersions is responsible for inferior polarity and it leads to weaker wettability of coatings [7, 8].

SFE plays a decisive role in the design of polyurethane biomaterials, e.g. antimicrobial polyurethane coatings [9] or blood-compatible materials [10, 11]. Optimization of the structure of polyurethanes for bone tissue engineering applications was described in [12]. For that purpose, aliphatic poly(ester-urethanes) were synthesised from poly(ε-caprolactone)diol with different molecular weights (530, 1,250 and 2,000 Da), cycloaliphatic diisocyanate and ethylene glycol as a chain extender. The hydrophilic performance of the polyurethane (PU) surface was characterised by the static water contact angle (Θ). The values of Θ on the PU surfaces decreased from 94.2° to 71.1° for the content of hard segments increasing from 22 to 77 wt.%. The smallest contact angle, which was indicative for the most hydrophilic structure, was measured for PU prepared from PCL 530, with 70% hard segments [12].

Taking into consideration the examples as above, we initiated a research programme within the possible modifications of the hydrophobic properties of elastomeric coats which had been derived from polyester–urethanes and polyether–urethanes. Said modifications were planned to be controlled solely by changes in the chain structures, i.e. they would result from the specific types of the raw materials employed: diisocyanates, polyols and chain extenders. There were no studies of that kind published in the scientific journals. Most reports, in which the hydrophobic performance of polyurethanes was mentioned, discussed that issue superficially only and from the viewpoint of the applicability of polyurethanes.

Therefore, the purpose of our study was to find some generalisation within that area. We refer to our previous papers in which we presented the possibility of modifying the SFE values in polyurethane ionomer coats [13–15]. We were prompted to undertake that kind of research inter alia by the findings presented in our earlier report [16]: the investigation with the use of wide angle X-ray scattering (WAXS) and AFM methods demonstrated that polyurethane coatings performed like elastomers and they contained up to 30% of crystalline phases which formed clearly separated rigid domains against the background of the amorphous phase. That condition was not easy to obtain while it might be essential for polarity of the coats which were formed with the use of those polymers. This paper, however, was aimed at classic polyurethane elastomers which had been produced in the solution polyaddition process, and test coats were prepared by simple evaporation from the apolar surface of poly(tetrafluoroethylene). In order to authenticate the calculations which involved numerous results collected (contact angle values) and to identify the physical interactions within the polyurethane chain structures, we calculated SFE data with the use of two complementary methods: by van Oss–Good and by Owens–Wendt.

## Experimental

### Reagents

Reagents used were as follows: methylene diphenyl 4,4′-diisocyanate (MDI); isophorone diisocyanate, [3-izocyanatomethyl –3,5,5- trimethylcyclohexyl isocyanate] (IPDI); polyoxyethylene glycols (*M*_n_ ≈ 600 g/mol; *M*_n_ = 2,000 g/mol) (POG; Aldrich) (that product was dried under vacuum in nitrogen, at 120 °C, for 2 h); poly(ε-caprolactone) diols (*M*_n_ ≈ 530 g/mol; *M*_n_ = 2,000 g/mol) (PCL) (Aldrich); and poly(ethyleneadipate) diol (*M*_n_ ≈ 1,000 g/mol) (PEA). All polyether and polyester reagents were dried under vacuum in nitrogen, at 120 °C, for 2–4 h.

*N*-Methyldiethanolamine (*N*-MDA), 2,2,3,3-tetrafluoro-1,4-butanediol (TFBD), 1,4-butanediol (BD), and 1,6-hexanediol (HD) (all reagents from Aldrich) were used as purchased. Dibutyl tin dilaurate (DBTDL) was purchased from Huntsman Performance Chemicals. Analytical reagents include dibutylamine, diiodomethane, formamide (from Aldrich) and redistilled water.

### Method for the synthesis of linear polyurethane

Polyurethanes were synthesised in a two-staged polyaddition process, in a glass stand composed of: three-necked flask, heating bowl, mechanical agitator, dropping funnel, thermometer, reflux condenser and nitrogen supply nozzle. The prepolymer was synthesised at stage 1 with the use of appropriate diisocyanates and polyols at the molar ratio of 2:1. The process was conducted at 60 °C for 2–3 h, in the presence of DBTDL as a catalyst which was added at 0.1 wt.% on polyether or polyester. The reaction was terminated when the concentration of free –NCO groups as established analytically was equal to that resulting from stoichiometric calculations. That stage may be presented by the following reaction:1where:

A: structure derived from polyol (POG, PCL or PEA)
B: structure derived from diisocyanate (MDI or IPDI).

It should be mentioned that the chemical constitution of so obtained BAB prepolymers is generally dependent on the type of diisocyanate (i.e. on reactivity of its functional groups and on the substitution effects) and on the reaction conditions (i.e. temperature, type and amount of catalyst), which may for example be favourable for the formation of allophanate structures [17]. Linear prepolymers were produced under the synthesis conditions as specified. They were then extended at the second stage with a suitable reactant (*N*-MDA, BD, HD or TFBD, respectively) in the solution in 1,4-dioxane, at the concentration of about 40 wt.%, with the molar ratio of the –NCO and –OH functional groups maintained at 1:1. The chain extension reaction was conducted at the reaction mixture boiling point (100–102 °C) until all free –NCO groups disappeared completely (2–3 h). The polymer chain extension process may be illustrated by the reaction:2where:

Q: structure derived from BD, HD, TFBD or *N*-MDA.

The final product obtained after extension of the prepolymer was the expected linear polyurethane. Its chain was composed of structural units which formed soft polyol segments A and hard urethane segments; the latter were compiled of diisocyanate-derived structural fragments B and chain extension fragments Q which were linked together with urethane bonds NH–CO–O– (x):3


Polyol segments derived from PCL or PEA in poly(ester-urethane) and PEO in poly(ether-urethane) made soft segments in the synthesised PUs. The hard segments, on the other hand, were composed of urethane segments—derived from diisocyanates which had not been converted at stage I or from prepolymer fragments with –NCO end groups and low molecular weight chain extenders.

**Fig. 1:**
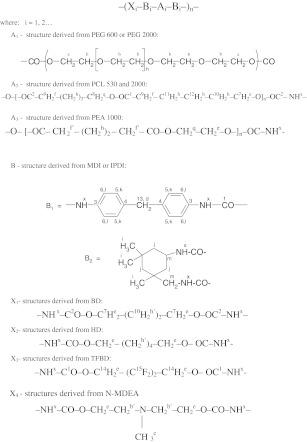
Structures of polyurethane chains in the synthesised polyurethanes

The chemical structures of the produced polyurethanes were presented in Table 1 and in Fig. 1. The reference coats were prepared by covering PTFE plates with the solution of linear polyurethane (about 40 wt.%) and conservative evaporation of 1,4-dioxane in a vacuum drier, at 80 °C, over 6 h, followed by additional conditioning by exposure to ambient air during 10 days.

**Table 1:** Chemical compositions of synthesised linear polyurethanes

Sample no.	Type of polyurethane	Type of diisocyanate	Type of polyol (molecular weight)	Type of chain extender	Fluorine content, wt.%
PU-1	Poly(ester-urethane)	MDI	PCL (530)	TFBD	6.01
PU-2		MDI	PCL (530)	BD	0
PU-3		MDI	PCL (2,000)	BD	0
PU-4		MDI	PEA (1,000)	*N*-MDA	0
PU-5		IPDI	PEA (1,000)	*N*-MDA	0
PU-6	Poly(ether-urethane)	MDI	POG (600)	TFBD	5.70
PU-7		MDI	POG (600)	HD	0
PU-8		MDI	POG (2,000)	BD	0
PU-9		MDI	POG (2,000)	*N*-MDA	0
PU-10		IPDI	POG (2,000)	BD	0
PU-11		IPDI	POG (2,000)	*N*-MDA	0

### Determination of –NCO group content

That analysis involved a well-known method, and dibutylamine was used in the tests. Excess of unreacted amine was titrated with the HCl solution, and bromophenol blue was used as an indicator [18].

### NMR spectroscopy

^1^H and ^13^C NMR spectra of the obtained polyurethanes (and additionally ^19^F NMR spectra for PU-1 and PU-2) were taken with the use of the FT NMR Bruker Avance 500^II^ spectrometer. The samples of coatings (i.e. produced cationomers) were dissolved in DMSO-d_6_/h–DMSO and the solutions with the concentration of about 0.2 g/dm^3^ were prepared. TMS was used as a standard. The proton spectra were employed additionally for the comparative polarity analysis of the cationomers which had no fluorine atoms in polymer chains. The study was based on the parameter *κ* which was defined especially for that purpose. That parameter was calculated from the values of integrated signals in ^1^H NMR spectra of polyurethanes. The following protons were distinguished: those representing polar (*I*_P_) and apolar (*I*_N_) structural fragments within the cationomer chains.

The factor *κ* was calculated as:

4 $$ \kappa {}_{{\exp }} = \frac{{{I_{\text{P}}}}}{{{I_{\text{P}}} + {I_{\text{N}}}}} \cdot 100\% $$

where:

5 $$ {I_{\text{P}}} = \sum\limits {{I_{{{{\text{P}}_n}}}} = {I_{\text{c}}} + 0,5({I_{\text{b}}} + {I_{{{\text{b'}}}}}) + {I_{\text{m}}} + {I_{\text{x}}}} $$

6 $$ {I_{\text{N}}} = \sum\limits {{I_{{{{\text{N}}_n}}}} = {I_{\text{i}}} + {I_{\text{j}}} + {I_{\text{h}}} + {I_{{{\text{h'}}}}} + {I_{\text{f}}} + {I_{{{\text{f'}}}}} + 0,5({I_{\text{b}}} + {I_{{{\text{b'}}}}}) + {I_{\text{d}}} + {I_{\text{k}}} + {I_{\text{l}}}} $$

For simplification, we accepted that CH_2_–O (b) and CH_2_–N (b′) groups had equivalent contributions of polar and nonpolar interactions (Table 3).

### IR spectra

IR spectra within 700–4,000 cm^−1^ for the obtained polyurethane coats were recorded with the use of the Nicolet 6700 FT-IR spectrophotometer and ATR technique.

### WAXS analysis

The WAXS investigations were performed at room temperature on the modified DRON-3-SEIFERT automated diffractometer. The radiation of CuKα and the nickel filter were employed. The operating conditions for the X-ray tube were as follows: 40 kV and 30 mA. X-ray diffraction patterns were taken within the range of 2Θ from 1° to 60°, with a scanning step equal to 0.02° and at the counting time of 10 s.

### Confocal microscopic analysis

The 3D NanoFocus optical measurement system is a compact package for the measurements and material analysis. Its high resolution (nanometric precision) is based on the innovative confocal multi-pinhole technology in combination with the piezo module. The lens used in the measurements sized between 1.6 × 1.6 and 260 × 260 μm. The image acquisition module from the NanoFocus system is a fast digital camera with the progressive scan technology, up to 55 fps, 512 × 512 pixel, 10 bit. The measurement pictures were taken for the object size of 320 × 320 μm with the magnification of ×50.

### Differential scanning calorimeter analysis

A differential scanning calorimeter from Mettler Toledo, type 822, was used to find the glass transition temperatures for soft segments, i.e. those derived from polyol structures, *T*_g1_, and for hard segments, i.e. those derived from urethanes and low molecular weight chain extenders, *T*_g2_. Each sample was subjected to the heating/cooling cycle within the temperatures from −50 °C up to 100 °C, at the rate of 10°/min.

### Surface roughness

The surface roughness grade of the synthesised coatings was determined by means of the MarSurf PS1 apparatus (from Mahr). The following parameters were measured to describe the surface roughness of the studied coats:

*R*_a_: arithmetic mean of the absolute values for *y* deviations of the profile from the average line *n*, over the elementary length *l*:

7 $$ {R_{\text{a}}} = \frac{1}{n}\mathop{\sum }\limits_{{i = 1}}^n \left| {{y_i}} \right| $$

8

*R*_z_: arithmetic mean of the absolute values for five highest peaks in the roughness profile (*y*_*pi*_) and five deepest pits in the roughness profile (*y*_*vi*_), over the elementary length *l*:

9 $$ {R_{\text{z}}} = \frac{1}{5}\left( {\sum\limits_{{i = 1}}^5 {\left| {{y_{{pi}}}} \right|} + \sum\limits_{{i = 1}}^5 {\left| {{y_{{vi}}}} \right|} } \right) $$

10


### Contact angle

Contact angles Θ were measured with the use of the method suggested by Zisman [19], i.e. by means of an optical goniometer with a digital camera installed in the axial direction of its lens. The liquid drops with the constant volume (about 3–5 μdm^3^) were applied to the surfaces of the studied samples with the use of a special micropipette. The samples were fixed on the stage of the goniometer. The measurements were taken at 21 ± 1 °C. The values of contact angles were found from the geometric analysis of pictures taken for liquid drops, which involved the use of our originally developed software *Kropla* for interpretation.

### Surface free energy

Physical parameters of the surface energy of a solid *γ*_*S*_ were found on the basis of the Owens–Wendt method and simultaneously on the basis of the van Oss–Good method. The Owens–Wendt model assumes that the surface free energy *γ*_*S*, *L*_ may be presented as a sum of two components [20]:

11 $$ {\gamma_{{S,L}}} = \gamma_{{S,L}}^d + \gamma_{{S,L}}^p $$

where:

$$ \gamma_{{S,L}}^d $$
surface energy connected with dispersion interactions,
$$ \gamma_{{S,L}}^p $$
surface energy connected with polar acid–base interactions.

Equation 11 is generally applicable both to a solid phase, and the subscript *S* is used then, and to a wetting liquid (standard liquid or tested liquid), with the subscript of *L*. The Owens–Wendt method was also convenient to us since it made it possible to evaluate the share of polar interactions in the total value of SFE, and thus, it was possible to refer the values obtained for *γ*_*S*_ to the ‘amounts’ of polar structures in cationomers (*κ*), as estimated from NMR spectra (Eq. 4)

The SFE value for solids (*S*) and for liquids (*L*) which interact with those solids should satisfy the Owens–Wendt equation:

12 $$ {\gamma_L} \cdot \frac{{1 + \cos \Theta }}{2} = \sqrt {{\gamma_S^d \cdot \gamma_L^d}} + \sqrt {{\gamma_S^p \cdot \gamma_L^p}} $$

where Θ is the experimentally found wetting angle between the liquid drop and the solid surface under investigation. So, wetting angles Θ were first measured for the surfaces of PU coatings with the use of two pairs of model liquids (water–diiodomethane and formamide–diiodomethane) with known parameters *γ*_*L*_, *γ*_*L*_^*d*^ and *γ*_*L*_^*p*^ (Table 2) [20]. Then, Eq. 12 was used to calculate the values *γ*_*S*_^*p*^ and *γ*_*S*_^*d*^ for the studied polyurethanes. The values of *γ*_*S*_ were calculated from Eq. 11.

**Table 2:** Surface properties of model measuring liquids [20]

Model measuring liquid	Surface free energy parameters [mJ/m^2^]						
	*γ*_*L*_	*γ*_*L*_^LW^	*γ*_*L*_^AB^	*γ*_*L*_^−^	*γ*_*L*_^+^	*γ*_*L*_^d^	*γ*_*L*_^p^
Water	72.8	21.8	51	25.5	25.5	21.8	51
Formamide	58.0	39.0	19.0	2.28	39.6	–	–
Diiodomethane	50.8	50.8	0	0	0	48.5	2.3

The van Oss–Good model assumes that the free surface energy *γ*_*S,L*_ may be presented as a sum of two components [21, 22]:

13 $$ {\gamma_{{S,L}}} = \gamma_{{S,L}}^{\text{LW}} + \gamma_{{S,L}}^{\text{AB}} $$

where:

$$ {\gamma}SLW $$
surface energy connected with long-range interactions (dispersion, polar and induction interactions)
γ_S_^AB^: surface energy connected with acid–base interactions, as results from the Lewis theory.

Let us use the symbol *γ*_*S*_^+^ for the component of *γ*_*S*_^AB^ which is responsible for the free surface energy of the Lewis acid, and the symbol *γ*_*S*_^−^ for the component representing the Lewis base. On the basis of the Berthelot theory, which assumes that interactions between molecules of different bodies located on a surface are equal to the geometric mean of interactions between molecules within each of those bodies, one can now formulate the following relations:

- For bipolar substances (liquids and surfaces of solids), which can be equivalent to synthesised PU—present in the form of aqueous dispersions or coatings:
  14 $$ \gamma_i^{\text{AB}} = 2\sqrt {{\gamma_i^{ + } \cdot \gamma_i^{ - }}} $$
- for nonpolar liquids and surfaces of solids (diiodomethane and PTFE):
  15 $$ \gamma_i^{\text{AB}} = 0 $$

(where: *i* = *S* – solid, *L* – liquid).

The SFE parameters for solids (*S*) and for liquids (*L*) which interact with those solids should satisfy the van Oss–Good equation:

16 $$ \sqrt {{\gamma_S^{\text{LW}} \cdot \gamma_L^{\text{LW}}}} + \sqrt {{\gamma_S^{ + } \cdot \gamma_L^{ - }}} + \sqrt {{\gamma_S^{ - } \cdot \gamma_L^{ + }}} = {\gamma_L} \cdot \frac{{1 + \cos \Theta }}{2}. $$

Wetting angles Θ were first measured for the surfaces of PU coatings with the use of three model liquids (water-diiodomethane-formamide) with known parameters of *γ*_*L*_, *γ*_*L*_^LW^, *γ*_*L*_^+^ and *γ*_*L*_^−^ (Table 2), and then, Eq. 16 was used to calculate the values of *γ*_*S*_^LW^, *γ*_*S*_^+^ and *γ*_*S*_^−^ for the studied polyurethanes. The values of *γ*_*S*_^AB^ were calculated from Eq. 14, while the values of *γ*_*S*_ were from Eq. 13.

### Mechanical properties

Mechanical investigations of the produced polyurethanes involved the testing machine: UTS 50 Modernizacja Zwick/Roell. Changes in strength parameters were recorded at the stretching speed of 10 mm/min, at the applied force of 1 N and at the travelling speed of the measurement module of 1 mm/min [23]. Special paddle-like test pieces were prepared with the dimensions as required.

Tensile strength (*R*_r_) was calculated from the formula:

17 $$ {R_{\text{r}}} = \frac{{{F_{\text{r}}}}}{{{A_{\text{o}}}}} $$

where:

*F*_r_: destructive force, (in newton)
*A*_o_: cross-section of test piece, perpendicular to the force direction, (in square millimetres).

18 $$ {A_{\text{o}}} = g \cdot b $$

where:

*g*: thickness of test piece (in millimetres)
*b*: width of test piece, (in millimetre)

Unit elongation (*ε*) was established as the length increment referred to the initial length of the test piece:

19 $$ \varepsilon = \frac{{\Delta {L_{\text{o}}}}}{{{L_{\text{o}}}}} \cdot 100\% $$

where:

*L*_o_: length of test piece (in millimetres)
*∆L*_o_: increment in length of test piece (in millimetres)

The Young’s modulus (*E*) was found as the ratio of the difference in stress values *σ*_2_ and *σ*_1_ to the difference in strain values:

20 $$ E = \frac{{{\sigma_2} - {\sigma_1}}}{{{\varepsilon_2} - {\varepsilon_1}}} $$

where:

*σ*_1_: stress, in megapascals, measured for the unit elongation *ε*_1_ = 0.05%,
*σ*_2_: stress, in megapascals, measured for the unit elongation *ε*_2_ = 0.25%.

## Results and discussion

### Chemical structures of synthesised polyurethanes

**Fig. 2:**
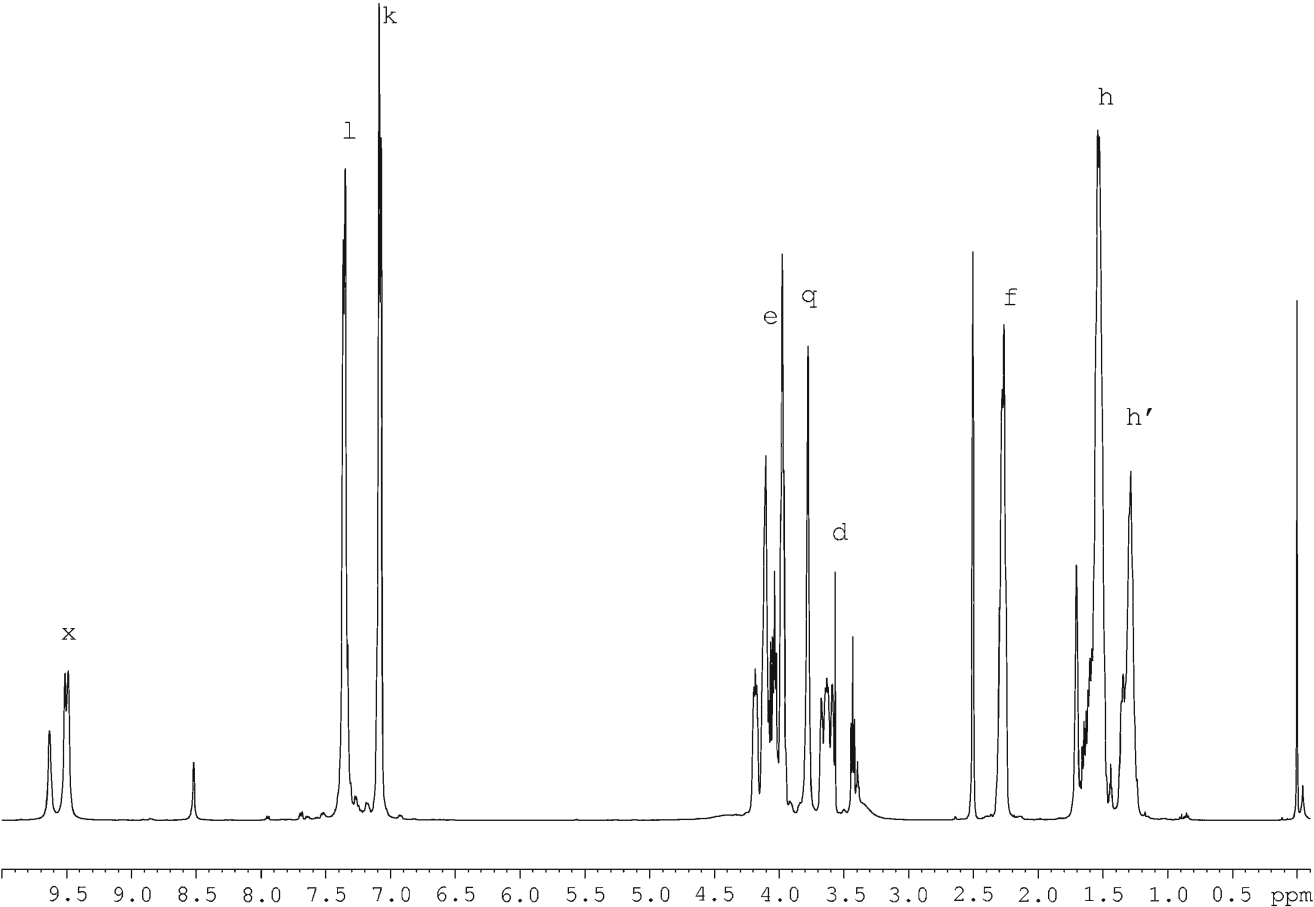
^1^H NMR spectrum of PU-2 poly(ester-urethane) sample

**Fig. 3:**
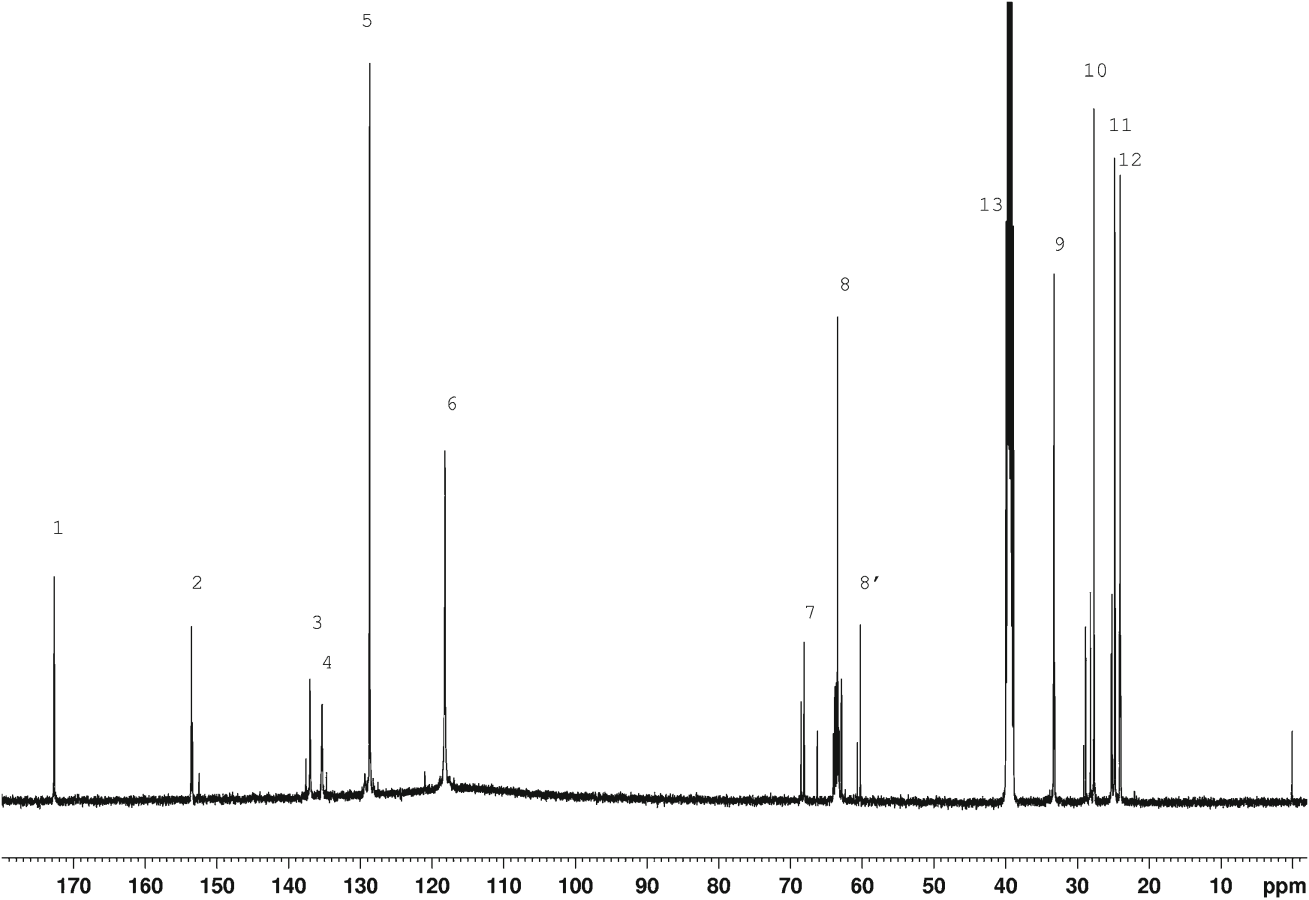
^13^C NMR spectrum of PU-2 poly(ester-urethane) sample

Chemical structures of synthesised polyurethanes were presented in Fig. 1. NMR spectroscopy confirmed the presence of signals for expected protons and carbon atoms. Figure 2 presents the exemplary ^1^H NMR spectrum for polyurethane PU-2, and Fig. 3 presents the ^13^C NMR spectrum for that polymer. The protons which are present in the structures of all synthesised polyurethanes were designated with letters (Fig. 2), while their chemical shift values *δ* were specified in Table 3, column 2. The carbon atoms which are present in the chemical structure of the exemplary sample of PU-2 were designated with letters, and the corresponding values of *δ* were attributed to them (Fig. 1, Table 4). ^1^H NMR spectra were additionally utilised to evaluate the polarity level of the structures studied, and a special parameter *κ*_exp_ was defined and calculated for that purpose.

**Table 3:** Analysis of signal integration in ^1^H NMR spectra of synthesised polyurethanes

Sample no.	*δ* (ppm)	Type of structural units	PU-2 (Fig. 2)	PU-3	PU-5	PU-7	PU-8	PU-9	PU-10	PU-11
Type of proton			Integration (conventional unit)							
*I*_i_	0.76–1.11	Nonpolar	–	–	1.705	–	–	–	6.3691	4.2847
*I*_j_	1.30–1.31				0.9545	–	–	–	0.1428	0.0970
*I*_h′_	1.29–1.45		0.9873	4.364	–	–	–	–	–	–
	1.21–1.61		–	–		2.3350	–	0.0436	–	
	1.46–1.70						0.3629	–	1.9119	
*I*_h_	1.53–1.71		2.4472	9.0773	–	–	–	–	–	–
	1.44–1.54			–	2.1527					
*I*_f_	2.26–2.30		0.9343	4.2588	–	–	–	–	–	–
*I*_f′_	2.45–2.68		–	–	–	–	–	–	–	–
	2.57–2.71				0.4544					
0.5I_b′_	3.33–3.80		–	–	0.1648	–	–	–	–	–
0.5 (*I*_b_ + *I*_b_′)	3.32–3.52		–	–	–	–	–	12.4141	–	
*I*_d_	3.57		0.1455	0.3408	–	1.2695	0.4814	0.5357	–	–
*I*_q_	3.78		0.4849	0.4988	–	–	–	–	–	–
	3.89				0.1970					
0.5*I*_b_	3.33–3.63		–	–	–	6.3178	12.6007	–	–	–
0.5 (*I*_m_ + *I*_b_)	3.07–3.66		–	–	–	–	–	–	21.6849	–
0.5 (*I*_m_ + *I*_b_ + *I*_b′_)	3.31–3.66		–	–	–	–	–	–	–	14.2606
*I*_k_ + *I*_l_	7.07–7.52		2.0082	2.0879	–	4.2742	2.0817	1.9884	–	–
*I*_N_			7.0074	20.6276	5.6284	14.1965	15.5267	14.9818	30.1087	18.6423
*I*_c_	2.24–2.25	Polar	–	–	1.1019	–	–	0.2829	–	0.4041
0.5I_b′_	3.45–3.61		–	–	–	–	–		–	
0.5 (*I*_m_ + *I*_b′_)_′_	3.57–3.60		–	–	0.1648	–	–	–	–	–
0.5*I*_b_			–	–	–	6.3178	12.6007	12.4141	–	–
0.5 (*I*_m_ + *I*_b_)			–	–	–	–	–	–	21.6849	–
0.5 (*I*_m_ + *I*_b_ + *I*_b′_)	3.01–3.66		–	–	–	–	–	–	–	14.2606
*I*_e_	3.92–4.19		1.8344	5.1812	1.0000	2.0916	0.8841	0.7459	1.5340	1.0000
	4.02–4.31		–	–	–	–				
*I*_x_	8.5–9.6		0.4951	0.4900	0.000	1.0396	0.5074	0.5195	0.0154	0.0485
*I*_P_			2.3285	5.6712	2.2667	9.4490	13.9922	13.9624	23.2343	15.7132
*κ*_exp_ (%)			24.94	21.56	28.71	39.96	47.40	48.24	43.56	45.74

**Table 4:** Interpretation of ^13^C NMR spectrum for PU-2 sample

Type of C nucleus (Fig. 1)	Chemical shift *δ*, ppm	
	PU-1	PU-2 (Fig. 3)
1	172.68	172.60
2	153.36; 153.52; 151.89; 152.05	152.46; 153.36; 153.50; 153.56
3	134.74; 135.17; 136.06; 136.15; 136.26; 136.35; 136.98; 137.06; 137.09; 137.62	137.02; 137.07; 137.58
4	134.74; 135.17; 135.28	134.75; 135.29; 135.35
5	128.74; 128.86; 128.89; 129.40	128.73
6	118.23; 118.55	118.22
7	68.11; 68.14; 68.53; 68.57	68.12; 68.14; 68.53; 68.57
14	66.28	
8	62.86; 62.91; 63.19; 63.30; 63.41; 63.78	62.86; 62.91; 63.19; 63.30; 63.41; 63.60; 63.78; 63.99
8′	59.29; 59.48; 59.66; 59.84	60.23; 60.67
15	58.46; 58.67; 58.88	
9	33.15; 33.18; 33.28; 33.33	33.16; 33.18; 33.28; 33.33
10	27.72; 28.16	27.72; 28.16; 28.80; 29.14
11	24.76; 24.81	24.77; 24.81; 25.19; 25.29
12	23.95; 24.00; 24.07	23.94; 23.96; 24.01; 24;08
13	~40	~40
–CH_3_ in DMSO	40.5	40.5

Such analyses were not conducted for PU-1 and PU-6. The synthesis of which involved TFBD, since—as results from our earlier research [24]—the presence of fluorine atom(s) in the polyurethane chain makes the most decisive factor for the polarity of a polymer coat. The presence of fluorine in polyurethane chains was confirmed by the ^19^F NMR spectrum of the PU-1 sample; the spectrum showed specific groups of signals: *δ* = −120.97; −121.73 to −122.88 and −123.09 ppm.

In order to find the experimental parameter *κ*_exp_, the integration values were analysed for all proton signals which were recorded within the NMR spectrum of a given sample. In particular, integrations of signals were distinguished for the protons which were present in functional groups and/or structural fragments with the polar and apolar character. Those calculations were itemised in Table 5. Independently, assuming the chain structure as per (3), the additional parameter of *κ*_theor_ was calculated:

21 $$ {\kappa_{\text{theor}}} = \frac{{\sum {n_i^{\text{polar}}} }}{{\sum {\left( {n_i^{\text{polar}} + n_i^{\text{apolar}}} \right)} }} $$

where:

*n*_*i*_^polar^: amount of protons in analysed polyurethane chain structures which were formally assumed as polar,
*n*_*i*_^apolar^: amount of protons in structures which were formally assumed as apolar.

**Table 5:** Calculation of polarity parameter *κ*_theor_ from the type and amount of protons in polyurethane chains 1–11 (Table 1)

Type of structural unit	Type of protons	Structural fragment derived		Polyurethane sample no.								
				PU-2	PU-3	PU-4	PU-5	PU-7	PU-8	PU-9	PU-10	PU-11
				Amount of protons								
Polar	–NH–CO–O–	MDI		4	4	4	–	4	4	4	–	–
		IPDI		–	–	–	4	–	–	–	4	4
	–CH_2_–NH–CO–O	IPDI		–	–	–	4	–	–	–	4	4
	≥CH–NH–CO–O–			–	–	–	2	–	–	–	2	2
	–CH_2_–O–CO–NH– *	A + BD + HD + TFBD + *N*-MDA		8	8	8	8	8	8	8	8	8
	O–CH_2_–CH_2_–O (½ total amounts)	POG 600		–	–	–	–	26.50	–	–	–	–
	O–CH_2_–CH_2_–O (½ total amounts)	POG 2000		–	–	–	–	–	90.20	90.20	90.20	90.20
	–O–[–OC–CH_2_–(CH_2_)_3_–CH_2_–O]_4.65_–OC–	PCL ‾*M*_n_ = 530		9.30	–	–	–	–	–	–	–	–
	–O–[−OC–CH_2_–(CH_2_)_3_–CH_2_–O]_17.54_–OC–	PCL *M*_n_ = 2,000		–	35.08	–	–	–	–	–	–	–
	–[–OC–(CH_2_)_4_–CO–O–CH_2_–CH_2_–O]_5.72_–(assumed ½)	PEA 1000		–	–	11.44	11.44	–	–	–	–	–
	(O–CH_2_–CH_2_)_2_N–CH_3_	*N*-MDA		–	–	4	4	–	–	4	–	4
	(O–CH_2_–CH_2_)_2_N–CH_3_	*N*-MDA		–	–	3	3	–	–	3	–	3
	Sum of protons in polar groups			21.30	47.08	30.44	36.44	38.50	102.20	109.20	108.20	115.20
Nonpolar	O–CH_2_–CH_2_ –O (½ total amounts)		POG 600	–	–	–	–	26.50	–	–	–	–
	O–CH_2_–CH_2_ –O (½ total amounts)		POG 2000	–	–	–	–	–	90.20	90.20	90.20	90.20
	–O–[–OC–CH_2_–(CH_2_)_3_–CH_2_–O]_4.65_–OC–		PKL ‾*M*_n_ = 530	9.30	–	–	–	–	–	–	–	–
	–O–[–OC–CH_2_–(CH_2_ )_3_–CH_2_–O]_4.65_–OC–		PKL ‾M_n_ = 530	27.90	–	–	–	–	–	–	–	–
	–O–[–OC–CH_2_–(CH_2_ )_3_–CH_2_–O]_17.54_–OC–		PKL M_n_ = 2000	–	140.32	–	–	–	–	–	–	–
	–[OC–CH_2_–(CH_2_ )_2_–CH_2_–CO–O–CH_2_–CH_2_–O]_5.72_–		PEA 1000	–	–	45.76	45.76	–	–	–	–	–
	–[–OC–(CH_2_)_4_–CO–O–CH_2_–CH_2_–O]_5.72_– (½ total amounts)		PEA 1000	–	–	11.44	11.44	–	–	–	–	–
	Ar–CH_2_–Ar		MDI	4	4	4	–	4	4	4	–	–
	Ar		MDI	16	16	16	–	16	16	16	–	–
	CH_3_–C		IPDI	–	–	–	18	–	–	–	18	18
	C–CH_2_–C		IPDI	–	–	–	12	–	–	–	12	12
	O–CH_2_–(CH_2_ )_4_–CH_2_–O		HD	–	–	–	–	8	–	–	–	–
	O–CH_2_–(CH_2_ )_2_–CH_2_–O		BD	4	4	–	–	–	4	–	4	–
	Sum of protons in apolar groups			61.20	164.32	77.20	87.20	54.50	114.20	114.20	124.20	120.20
*κ*_theor_				25.82	22.24	28.28	29.47	41.40	47.23	48.88	46.56	49.07
*κ*_exp_				24.94	21.56	–	28.71	39.96	47.40	48.24	43.56	45.74

**Fig. 4:**
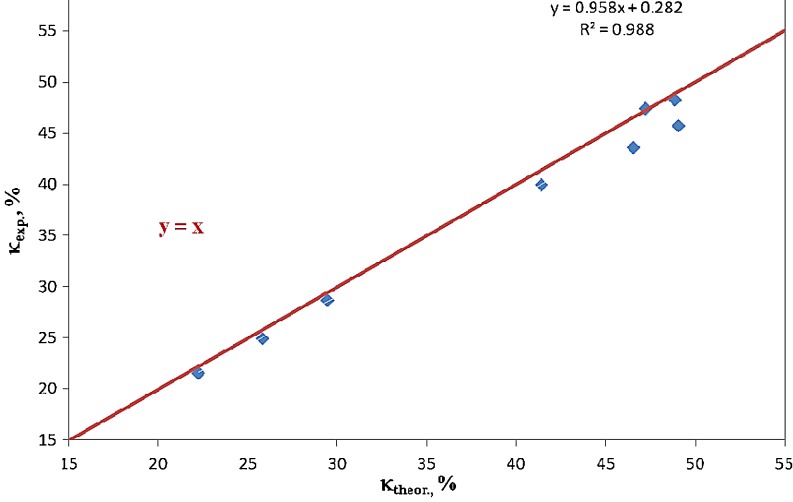
Graphical presentation of relationship between *κ*_theor_ and *κ*_exp_ polar parameters

Alike for proton signals in NMR spectra, CH_2_–O and CH_2_–N structures were assumed to bring similar contributions to polar and apolar (dispersive) interactions. The results of those calculations were specified in Table 4. The calculation procedure as adopted for the parameter *κ*_theor_ made it possible to give consideration to differences in molecular weights of polyols which had been used in the synthesis: POG, PCL and PEA. For that purpose, the structures of those polyols were attributed the average numbers of structural repeating units (mers) which resulted from their molecular weight values. Thus, the parameter *κ*_theor_ could be used to evaluate the effect of polar interactions in the synthesised polyurethanes solely from the structural viewpoint. If chemical structures of obtained polyurethanes were perfectly in line with (3), the points with coordinates *κ*_theor_ and *κ*_exp_ should be situated on the straight line *y = x* as shown in Fig. 4 which presents the points with actual coordinates. The obtained regression equation *y = 0.958x + 0.282* demonstrates that all specified points for which coordinate values were established within the real values of *κ*_theor_ are located below the straight line *y = x*; hence, fewer polar structures are present in the synthesised polyurethanes than one could expect. That proves a systematic discrepancy between the chemical compositions of PUs as expected from stoichiometry and the compositions which are actually available from the synthesis. It is important since polar structures are formed generally as late as in the polyaddition process (e.g. bands of protons designated as *e* or *x*), while formally apolar structures appear even in isocyanate or hydroxyl parent substances. The parameters *κ*_exp_ and *κ*_theor_ were useful in the further part of the study to analyse the effect of the PU structure itself on SEP parameters of the polymer coating obtained from that PU.

**Fig. 5:**
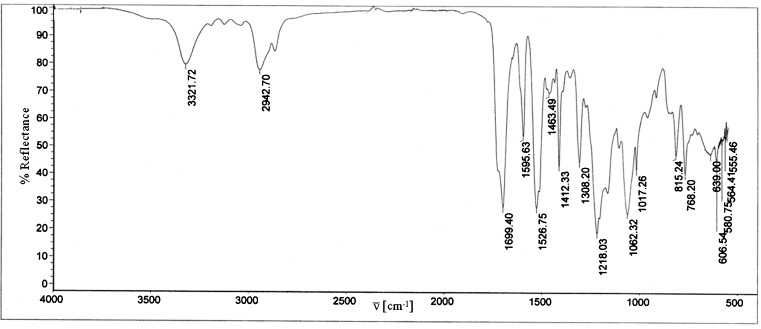
IR spectrum of PU-2 poly(ester-urethane) sample

IR spectra confirm complete conversion of parent substances and formation of structures which correspond to adequate polyurethanes. Figure 5 for example presents the IR spectrum of polyurethane PU-2; bands which are typical for PU can be seen in that spectrum (Table 6).

**Table 6:** Interpretation of FT IR spectrum of PU-2 sample

$\mathop{\nu }\limits^{ - }$, (Fig. 5) (cm^−1^)	Type of vibrations
3,322	Stretching vibrations, N–H groups in urethanes
2,943	Symmetric stretching vibrations in CH_2_ groups
1,699	C=O stretching vibrations for first amide band
1,596	C–C vibrations in benzene ring
1,527	Bending vibrations in N–H groups for second amide band
1,463	CH_2_ bending vibrations in aliphatic chains
1,413	CH_2_ vibrations in CH_2_–CO– groups
1,308	CH_2_ bending vibrations CH_2_
1,218, 1,062, 1,017	C–O–C stretching vibrations in urethane, ethers and esters
815	C–H bending vibrations in 1,4-substituted benzene ring
768, 639	C–H bending vibrations in CH_2_ groups

### Phase structures and mechanical properties of synthesised polyurethanes

From our earlier research, correct interpretation of FSE for polymer coats must give consideration not only to the chemical structure but to the phase structure as well which in case of polyurethane plastics may be much diversified and may comprise considerable amounts of crystalline phase. Polar and dispersion interactions between soft and hard segments, and in particular the presence of hydrogen bonds which involve the –NH– donors as present in urethane groups, and acceptors with the nature of carbonyl groups in urethanes and polyesters, as well as oxygen atoms in urethane, ether and ester groups, are of crucial importance in that instance. Those interactions may arrange the polyurethane structures orderly so that the domains will form regular segments of hard crystalline phase which will be enclosed in the dispersed amorphous phase [16].

**Fig. 6:**
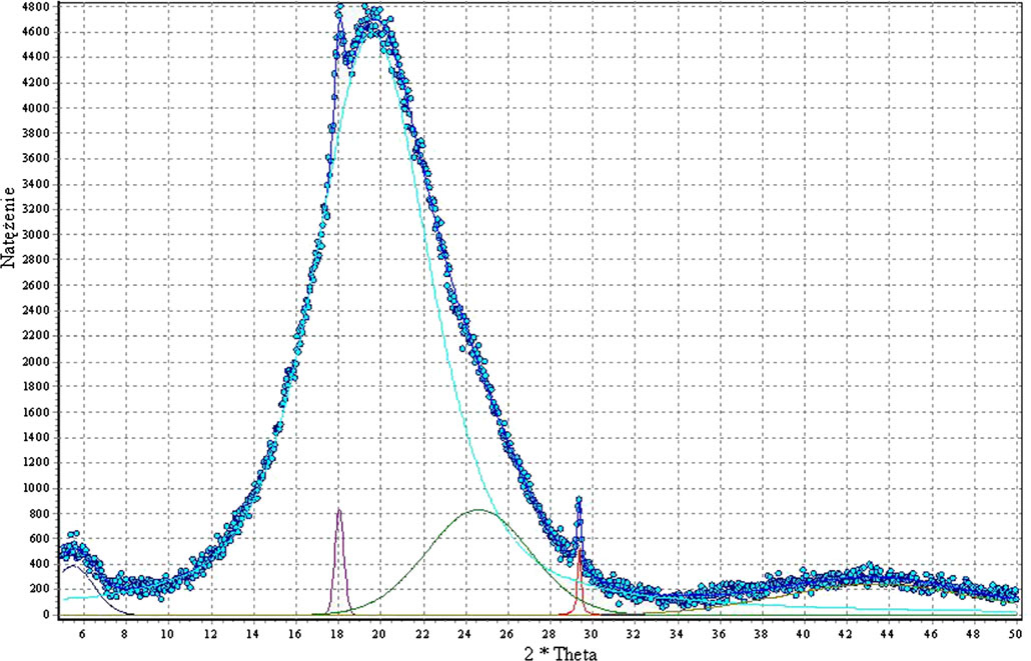
WAXS diffraction pattern for PU-2 poly(ester-urethane) sample

WAXS and DSC analyses were performed in order to investigate the nature of the arrangement in synthesised polyurethanes. The WAXS tests show that our polyurethanes are generally amorphous; they contain a small fraction of crystalline phase which, however, is hard to measure quantitatively. Figure 6 presents an exemplary WAXS diffraction pattern of PU-2 sample. That profile was analysed (Table 7) to learn that the amorphous structure is encountered in this case, and two very narrow peaks may represent a small amount of more orderly structures, most probably low molecular weight ones.

**Table 7:** Interpretation of WAXS diffraction patterns for PU-2 sample

2 theta, °	*d* ^a^, $\mathop{A}\limits^{\text{o}}$	FWHM^a^	*A* ^a^
5.5	16.06	2.3	941
18.0	4.92	0.5	445
19.6	4.53	6.5	37,965
24.6	3.62	5.7	5,073
29.4	3.04	0.3	191
43.4	2.09	10.8	2,588

^a^ *d* interplanar spacing, *FWHM* half-intensity width, *A* peak area

**Fig. 7:**
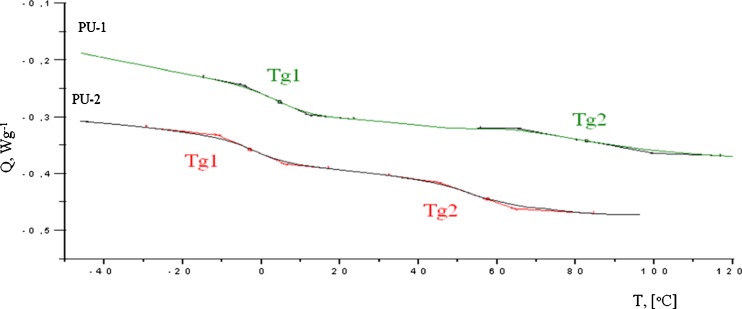
DSC curves for (**a**) PU-1 and (**b**) PU-2 polyurethane coatings

For comparison, Fig. 7 shows DSC thermograms for PU-1 and PU-2 samples. Which is specific is two glass transition values *T*_g1_ < 0 for soft polyol segments and *T*_g_ > 0 (Table 8) corresponding to hard urethane segments. Clear separation of those two phase transition regions is indicative for complete separation of domains which are formed of soft and hard segments, respectively. It is apparent, moreover, that the addition of fluorine increases temperatures for both those phase transitions. That should be accounted for by the increased share of fluorine-containing hard segments which consequently improves dispersion interactions. The results of mechanical tests for coatings, as provided in Table 8, confirm that the synthesised materials are elastomers and their strength at the level of about 40 MPa is sufficient for protective coatings. Hence, measurements of contact angles for those coats are justified. For regularity, we would like to mention that the coats turned out thermally stable up to about 250 °C (start of mass loss as observed by the TG method). The presence of fluorine (about 6 wt.%) improved thermal resistance considerably. The fastest degradation point moved towards higher temperatures for PU-1 sample in relation to PU-2 (*T*_max_ in DTG curves) by 12°. That increase in *T*_max_ for PU-6 and PU-8 samples amounted to 7°.

**Table 8:** Thermal and mechanical properties of same synthesised poly(ester urethanes)

Properties	PU-1 (with fluorine)	PU-2
Glass transition		
For soft segments; *T*_g1_, °C	4.7	−4.4
For hard segments; *T*_g2_, °C	83.8	58.1
*T*_ax_, °C	343	331
Mass loss at *T*_max,_ %	51.52	40.43
*R*_r_, MPa	4.08	6.72
$\frac{{\Delta L}}{{{L_{\text{o}}}}}$, %	22.8	66.4
*E*, MPa	37.4	45.9

**Fig. 8:**
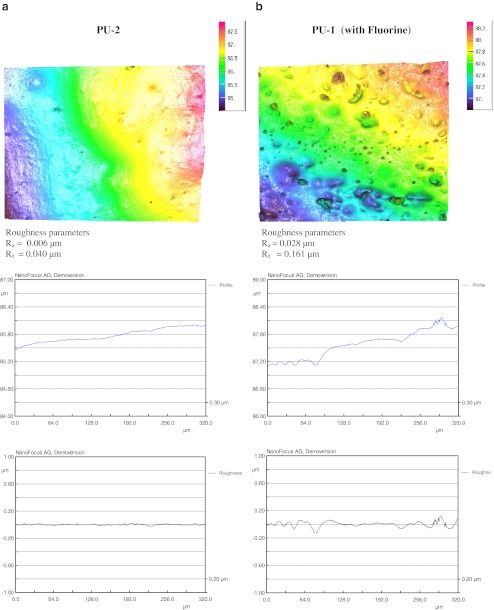
Images for coating obtained from PU-2 (**a**) and PU-1 (**b**), recorded under the confocal microscope

The shape of the surface and its roughness are essential if the measured contact angles and their interpretation are to be correct. In order to get to know about that condition, a confocal microscope was employed in the study. Figure 8 presents the analytical results and the roughness profiles for exemplary samples of PU-1 and PU-2, as well as the parameters *R*_a_ and *R*_z_ (Table 8). As can be seen, the coats which contained fluorine offered higher surface roughness values. Pursuant to interpretation in [24], that might result from the trend of fluorine to migrate towards the air/solid coat interface. Unfortunately, our latest studies with the use of the photoelectron spectroscopy technique have not confirmed that idea so far. Nevertheless, the observed surface is homogeneous within the base area of the liquid drop and makes it possible to obtain reliable measurements of contact angles.

### Surface free energy

The results of SFE measurements were presented in Table 9. The observed differences in calculations of free surface energy components by vO–G and O–W methods should be recognised as small in the light of accuracy and precision of measurements of the contact angle values. The results from the vO–G method suggest that it is long-range interactions *γ* ^LW^ to be decisive for the value of SFE, while acid–base interactions *γ* ^AB^ are negligible. The O–W method, on the other hand, shows that dispersion interactions *γ* ^d^ are more important within LW interactions for the evaluation of polarity of polyurethane coatings. It should hence be assumed that the parameter *κ*_exp_, as calculated with the use of NMR spectra and as defined by the formula (4), represents more general LW interactions. The synthesised coats are generally characterised by the values *γ*_*S*_ > 35 mJ/m^2^, i.e. they are polar materials in general. Definitely less polar are the coats which contain fluorine: PU-1 and PU-6, for which γ_*S*_ ≈ 30 mJ/m^2^. That value means a considerable reduction in SFE. We expected on the basis of the parameters *κ*_theor_ and *κ*_exp_ that the replacement of the chain extender BD with HD should reduce somewhat the SFE value of PU-7 in relation to PU-8. It turned out, however, that the expected effect was concealed by the use of polyether POG with *M*_n_ = 2,000 in the synthesis of PU-8. On the other hand, the increasing molecular weight of PCL (samples PU-2 and PU-3) results in a clear, although not so high, reduction in SFE, which can also be observed when comparing the parameters *κ*_theor_ and *κ*_exp_ (Table 5). The SFE of poly(ester-urethanes) is clearly lower than that for poly(ether-urethanes) which have been produced from the same diisocyanate and from polyol with the similar molecular weight (samples PU-2 and PU-8 versus PU-4 and PU-11). No increase in SFE was observed when BD as the chain extender was replaced by a more polar material, i.e. amine *N*-MDA (PU-8 and PU-9), which could be suggested by the values of *κ*_exp_ and *κ*_theor_. Our earlier analyses [25] show that more hydrophobic *N*-alkylamines may be needed to reduce the polarity of polyurethane coats. Yet, the quoted paper presented the research within SFE of coatings which were obtained from more complex polyurethane cationomers, in which *N*-MDA had a much more important function than just a chain extender. It should be finally mentioned that polyurethanes obtained from aromatic diisocyanate MDI (PU-4 and PU-8) turned out more hydrophobic coats than their structural analogues which had been synthesised with the use of IPDI (PU-5 and PU-10).

**Table 9:** SFE components for linear poly(ester-urethane) and poly(ether-urethane) coatings, estimation by Owens–Wendt and van Oss–Good methods

Polyurethane sample no. by Table 1	Type of polyurethane	van Oss–Good method by formamide–diiodomethane–water contact angles, [mJ/m^2^]			Owens–Wendt method by formamide–diiodomethane contact angles, [mJ/m^2^]			Owens–Wendt method by water–diiodomethane contact angles, [mJ/m^2^]		
		*γ*_*S*_	*γ*_*S*_^LW^	*γ*_*S*_^AB^	*γ*_*S*_	*γ*_*S*_^d^	*γ*_*S*_^p^	*γ*_*S*_	*γ*_*S*_^d^	*γ*_*S*_^p^
PU-1	Poly(ester-urethane)	27.28	27.20	0.08	27.28	26.58	0.695	27.55	27.27	0.281
PU-2		42.09	41.53	0.56	42.29	40.47	1.823	41.96	37.90	4.063
PU-3		39.75	37.70	2.05	39.09	33.04	6.050	39.87	31.48	8.39
PU-4		37.74	36.89	0.85	38.25	37.98	0.271	38.46	33.49	4.959
PU-5		41.03	37.02	4.01	40.55	33.05	7.497	41.42	32.38	9.033
PU-6	Poly(ether-urethane)	21.47	21.44	0.03	22.21	22.19	0.02	22.73	22.72	0.01
PU-7		42.37	42.34	0.03	44.11	44.10	0.01	43.87	43.84	0.03
PU-8		33.19	32.91	0.28	38.54	38.39	0.15	38.55	38.41	0.14
PU-9		30.39	28.99	1.40	33.50	33.07	0.43	33.18	31.60	1.58
PU-10		39.45	37.52	1.93	45.28	36.91	8.37	–	–	–
PU-11		37.77	35.57	2.20	42.17	35.70	6.47	–	–	–

## Conclusions

The study demonstrates that there are practical relations between the chemical structures of linear polyurethanes and free surface energy of the elastomeric coats obtained from those materials, with the amorphous character. The values of FSE can be controlled within 33–45 mJ/m^2^ by taking MDI or IPDI as well as polyethers and polyesters with molecular weights of 600–2,000 g/mol for the synthesis of polyurethanes. Definite reduction in SFE down to about 30 mJ/m^2^ can be obtained by adding fluorine in the form of TFBD as a chain extension agent to urethane–isocyanate prepolymers produced at the initial polyaddition stage.

The SFE values were calculated with the use of two methods: by van Oss–Good and by Owens–Wendt, based on contact angles as measured with the use of properly selected model liquids. Additionally, polarity was studied by the ^1^H NMR method. That research demonstrated that polarity of linear polyurethanes would be affected to the highest extent by long-range interactions (dispersion and polar interactions). These comprise inter alia the interactions which result from the presence of hydrogen bonds. The study confirmed that the nature of the test coat surface—its mechanical strength and surface roughness—was essential for the correct determination of contact angle values with the use of an optical goniometer.
